# Feasibility and acceptability of community-based psychosocial interventions delivered by nonspecialists for perinatal common mental disorders: A systematic review using an implementation science framework

**DOI:** 10.1017/gmh.2025.10010

**Published:** 2025-05-26

**Authors:** Prasansa Subba, Pragya Shrestha, Atif Rahman, Nagendra Luitel, Ahmed Waqas, Siham Sikander

**Affiliations:** 1 Department of Primary Care and Mental Health, University of Liverpool, Liverpool, UK; 2 Research Department, Transcultural Psychosocial Organization Nepal, Kathmandu, Nepal; 3 Department of Public Health, University of Copenhagen, Copenhagen, Denmark; 4 Faculty of Buddhist Studies, Lumbini Buddhist University, Lumbini, Nepal; 5 Human Development Research Foundation, Islamabad, Pakistan

**Keywords:** perinatal depression, perinatal anxiety, psychosocial intervention, nonspecialists, implementation science

## Abstract

Task sharing is endorsed as one of the strategies to address the treatment gap in common perinatal mental health conditions. There is a well-established body of evidence on the effectiveness of psychological interventions delivered by nonspecialist health workers (NSHWs); however, there is a dearth of evidence documenting factors determining the feasibility, acceptability and sustainability of integrating and implementing these interventions. This systematic review aims to synthesize the implementation outcomes and implementation process of NSHWs-delivered psychological interventions for the management of perinatal depression and anxiety using Proctor’s implementation science framework outlining eight constructs: feasibility, acceptability, appropriateness, adoption, cost, fidelity, penetration and sustainability. We searched PubMed, Web of Science and Cochrane Center Register of Controlled Trials for studies published in English and between 2000 and 2022 using search terms under five broad categories: (a) “perinatal”; (b) “common mental disorders”; (c) “psychological interventions”; (d) “nonspecialist” and (e) “implementation outcomes.” Secondary publications were also hand-searched for data extraction. Two authors independently reviewed abstracts and full-text articles. Data for included articles were extracted using a standard data extraction sheet. A narrative synthesis of qualitative evidence was conducted. Initial searches identified 885 articles of which full text of 128 articles were screened for eligibility, with 56 studies meeting the inclusion criteria. Out of the eight constructs of Proctor’s framework, “feasibility,” “acceptability,” “appropriateness” and “fidelity” were the most evaluated outcomes. None of the studies reported “penetration” and very few reported “sustainability,” “adoption” or “cost.” None of the studies used any implementation science framework for the study evaluation. Despite the well-established evidence on the effectiveness of psychosocial interventions for perinatal depression and anxiety by NSHWs, these interventions are rarely adopted into the health system. More studies applying systems thinking are needed to explore facilitators, barriers and mechanisms for integrating interventions in the health system. Using implementation science frameworks to design, plan, execute and evaluate psychosocial interventions by NSHWs can address this gap in evidence.

## Graphical abstract

**Figure:**
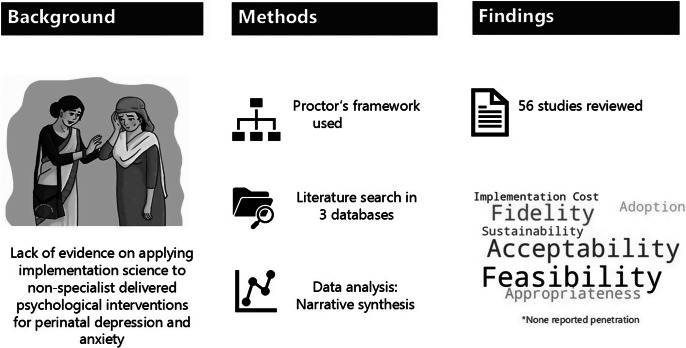

## Impact statement

This review synthesizes evidence on the implementation of psychological interventions for perinatal depression and anxiety delivered by nonspecialist health workers (NSHWs). Using Proctor’s framework, it highlights the successes, challenges and processes involved in these interventions offering insights for policymakers, healthcare administrators and practitioners to improve perinatal mental health programs. The review finds that NSHWs can deliver psychological intervention effectively if they are well-trained, supervised and properly incentivized. These interventions are more successful when they fit well with the local culture and integrated within the existing system. However, there is a critical gap in understanding the larger systems that affect the long-term success of these interventions. The review highlights the need for further research on how these programs can be integrated and sustained within the system.

## Introduction

Depression and anxiety are the most common perinatal (pregnancy up to 1 year postnatal) mental disorders (Waqas et al., 2022). Approximately 15% and 25% of women suffer from perinatal anxiety and depression and the burden is higher in low- and middle-income countries (LMICs) compared to high-income countries (HICs) (Nielsen-Scott et al., 2022; Mitchell et al., 2023). Perinatal mental disorders are associated with maternal suicide, poor uptake of health services, delayed social, emotional and cognitive development in infants, and marital discord (Dagher et al., 2021; Kroh and Lim, 2021; Wang et al., 2021; Stewart and Payne, 2023). Despite its debilitating effects on the woman, her infant and her social relationships, detection and treatment of perinatal depression remains a challenge (Gelaye et al., 2016). Evidence suggests that more than 80% of women with perinatal depression are out of care (Cox et al., 2016) and less than 40% intend to seek help (Daehn et al., 2022). This “treatment gap,” the gap between the need and access to treatment, is more prominent in marginalized populations such as women in rural areas, from ethnic minorities, or with poor socioeconomic status ( Stirling et al., 2001; Price and Proctor, 2009; Prady et al., 2021).

Challenges pertaining to the treatment gap can be broadly categorized into demand and supply-side challenges. Lack of awareness about depression, its treatment options, treatment availability, stigma, time constraints and the practice of “wait and get it over naturally” are common barriers impeding women to seek help (Dagher et al., 2021; Iturralde et al., 2021). Further, poor investment in mental health, scarcity of skilled and trained human resources, ill-equipped health facilities, stigma and lack of health professionals’ awareness contribute to the expanding treatment gap (Lasater et al., 2017; Dagher et al., 2021). The World Health Organization (WHO) (2021) reports that 50% of the world’s population lives in a place where there is less than one psychiatrist for 100,000 population. The WHO advocates for a task-sharing approach whereby expert knowledge and skills are transferred to nonspecialist health workers (NSHWs) (WHO, 2016). Psychological interventions are a first-line treatment recommended for perinatal depression. There is a well-established evidence base that shows psychological interventions delivered by NSHWs are effective both at preventing (Prina et al., 2023) and treating perinatal depression (Singla, Lawson, et al., 2021), but they do not adequately address the questions of *“how”* interventions can be successfully integrated and adopted across diverse contexts.

A review by Munodawafa (2018) discusses the context and mechanisms of successful implementation of interventions for perinatal depression. Additional evidence on intervention content for perinatal depression and its delivery in LMICs (Chowdhary et al., 2014) and HICs (Singla, Lawson, et al., 2021) also exists; however, a combined global evidence on the evaluation of these interventions using implementation science constructs is still lacking. As NSHWs continue to be an important cadre for delivering services for perinatal depression, it is important to understand how best they can be mobilized. Proctor et al (2011) have proposed eight constructs for documenting implementation outcomes, namely: acceptability, adoption, appropriateness, feasibility, fidelity, implementation cost, penetration and sustainability. The current systematic review thus aims to synthesize evidence on the implementation process of NSHW-delivered psychosocial interventions for the management of perinatal depression and anxiety, as well as implementation outcomes based on the Proctor’s framework (Proctor et al., 2011). The findings from this review will be valuable to policymakers, practitioners and academics working on task-sharing interventions to address perinatal mental health concerns.

## Methods

The protocol for this review was registered in the National Institute for Health Research with the PROSPERO registration No. CRD42022306566 on March 10, 2022. This systematic review followed the Preferred Reporting Items for Systematic Review guidelines for reporting (Page et al., 2021).

### Search strategy

The first (PS1) and second author (PS2) performed the search in three databases: PubMed, Web of Science and Cochrane Center Register of Controlled Trials. Search strategies were developed for each database using terms for five broad responses: “perinatal,” “common mental disorders,” “psychological interventions,” “nonspecialist” and “implementation” and filtered by date (1 January 2000 and 1 January 2022) (see Table 1). Full search strategy tailored to each database can be found in Supplementary File 1.

**Table 1.** Search strategy adapted for *PubMed* database

	Concept	Keywords
Population	Perinatal	(antenatal[Title/Abstract] OR antepartum[Title/Abstract] OR pregnan*[Title/Abstract] OR postpartum[Title/Abstract] OR postnatal[Title/Abstract] OR maternal[Title/Abstract] OR perinatal[Title/Abstract] OR peripartum[Title/Abstract] OR postpartum period[MeSH Terms])
Condition	Common mental disorders	(“depressive disorder*”[Title/Abstract] OR “depression”[Title/Abstract] OR “depress*”[Title/Abstract] OR “anxiety”[Title/Abstract] OR “anxiety disorder*”[Title/Abstract] OR “anxi*”[Title/Abstract] OR “common mental disorder*”[Title/Abstract] OR “common mental health problem”[Title/Abstract] OR “anxiety disorder” [MeSH Terms] OR “depressive disorder”[MeSH Terms] OR “depression, postnatal”[MeSH Terms])
Intervention	Psychological interventions	(“psychosocial counseling”[Title/Abstract] OR psychoeducat*[Title/Abstract] OR “non-pharmacological”[Title/Abstract] OR psychotherapy[Title/Abstract] OR “psychological therapy”[Title/Abstract] OR “group therapy”[Title/Abstract] OR “group counseling”[Title/Abstract] OR “group counseling”[Title/Abstract] OR “individual counseling”[Title/Abstract] OR “individual counseling”[Title/Abstract] OR “group session*”[Title/Abstract] OR “non-directive counseling”[Title/Abstract] OR “non-directive counseling”[Title/Abstract] OR “comprehensive psychosocial intervention*” [Title/Abstract] OR “multifaceted intervention*”[Title/Abstract] OR “integrated mental health service*”[Title/Abstract] OR “multicomponent intervention*”[Title/Abstract] OR “multidimensional intervention*”[Title/Abstract] OR “holistic intervention*” [Title/Abstract] OR “community based intervention*”[Title/Abstract] OR “cognitive behavioral therapy”[Title/Abstract] OR “dialectical behavior therapy”[Title/Abstract] OR “interpersonal therapy”[Title/Abstract] OR “relational therapy”[Title/Abstract] OR “talk therapy”[Title/Abstract] OR “psychosocial intervention”[MeSH Terms] OR “cognitive behavioral therapy”[MeSH Terms] OR “Dialectical Behavior Therapy”[MeSH Terms] OR “Interpersonal Psychotherapy”[MeSH Terms])
	Nonspecialist	(“nonspecialist*”[Title/Abstract] OR “task shar*”[Title/Abstract] OR “task shift*”[Title/Abstract] OR “community health worker*”[Title/Abstract] OR “lay health worker*”[Title/Abstract] OR “peer volunteer*”[Title/Abstract] OR “community volunteer*”[Title/Abstract] OR “health worker*”[Title/Abstract] OR “barefoot doctor*”[Title/Abstract] OR “psychosocial worker*”[Title/Abstract] OR “psychosocial counselor*”[Title/Abstract] OR “specially trained”[Title/Abstract] OR “nurse*”[Title/Abstract] OR “village health worker*”[Title/Abstract] OR “community health aide”[Title/Abstract] OR “community health workers”[MeSH Terms])
Outcomes	Implementation outcomes	(“clinical competence”[MeSH Terms] OR “program evaluation”[MeSH Terms] OR “feasibility”[Title/Abstract] OR “acceptability”[Title/Abstract] OR “evaluation”[Title/Abstract] OR “program evaluation”[Title/Abstract] OR “program”[Title/Abstract] OR “fidelity”[Title/Abstract] OR “implementation”[Title/Abstract] OR “practice”[Title/Abstract] OR “reach”[Title/Abstract] OR “penetration[Title/Abstract] OR “train*”[Title/Abstract] OR “clinical mentor*”[Title/Abstract] OR “clinical supervision”[Title/Abstract] OR “clinical competenc*”[Title/Abstract] OR “competenc*”[Title/Abstract] OR “attitude*”[Title/Abstract] OR “perception*”[Title/Abstract] OR “view*”[Title/Abstract] OR “behavior*”[Title/Abstract] OR “sustainab*”[Title/Abstract] OR “facilitator*”[Title/Abstract] OR “barrier*”[Title/Abstract])

### Screening

Two reviewers, PS1 and PS2, independently screened the titles and abstracts of studies identified through the database search. The full text of each article was then reviewed for eligibility.

### Data extraction

Data relevant to this review were extracted from selected papers into a Microsoft Excel spreadsheet. In the first step, the selected papers were evenly distributed between the two reviewers, PS1 and PS2. Each reviewer independently extracted relevant information and categorized it under the following headings: author, year, setting, design, intervention details, delivery agents, training, supervision, feasibility, acceptability, fidelity, barriers, facilitators, appropriateness, adoption, implementation cost, penetration and sustainability. In the subsequent step, PS1 and PS2 cross-reviewed each other’s data extraction tables to verify their accuracy and completeness. Any disagreements between the reviewers were discussed with AW.

### Quality assessment

PS1 appraised all studies and discussed any confusion with AW. The Critical Appraisal Skills Program (CASP) checklist was used for qualitative studies (Critical Appraisal Skills Programme, 2018). The CASP checklist examines methods, study design, positionality, data collection and analysis procedures where studies are rated “yes,” “no,” “insufficient” or “not applicable.” For quantitative and mixed-method studies, an assessment tool designed and used by Liu et al. (2019) was used. Studies were rated as “yes,” “no,” “partially,” “unclear” or “not applicable” under domains such as planning, design and conduct and reporting stages. Both the checklists do not use any quantitative scoring system.

### Data synthesis

Narrative synthesis was used to synthesize data on intervention implementation (Popay et al., 2006). We initiated a preliminary synthesis of the data as per the eight constructs of Proctor’s framework (Proctor et al., 2011) and explored relationships based on the intervention characteristics and study design. Where information was missing under certain outcomes, additional articles from the same studies were examined. Six additional articles (Glavin et al., 2010a; Segre et al., 2010; Segre et al., 2015; Lund et al., 2020; Davies et al., 2022; Yator et al., 2022) related to the included studies (Glavin et al., 2010b; Brock et al., 2017; Boisits et al., 2021; Yator et al., 2021) were reviewed for additional data. This review focused on the implementation process outcomes; hence, a meta-analysis was not conducted.

## Results

### Study selection

**Figure 1.**
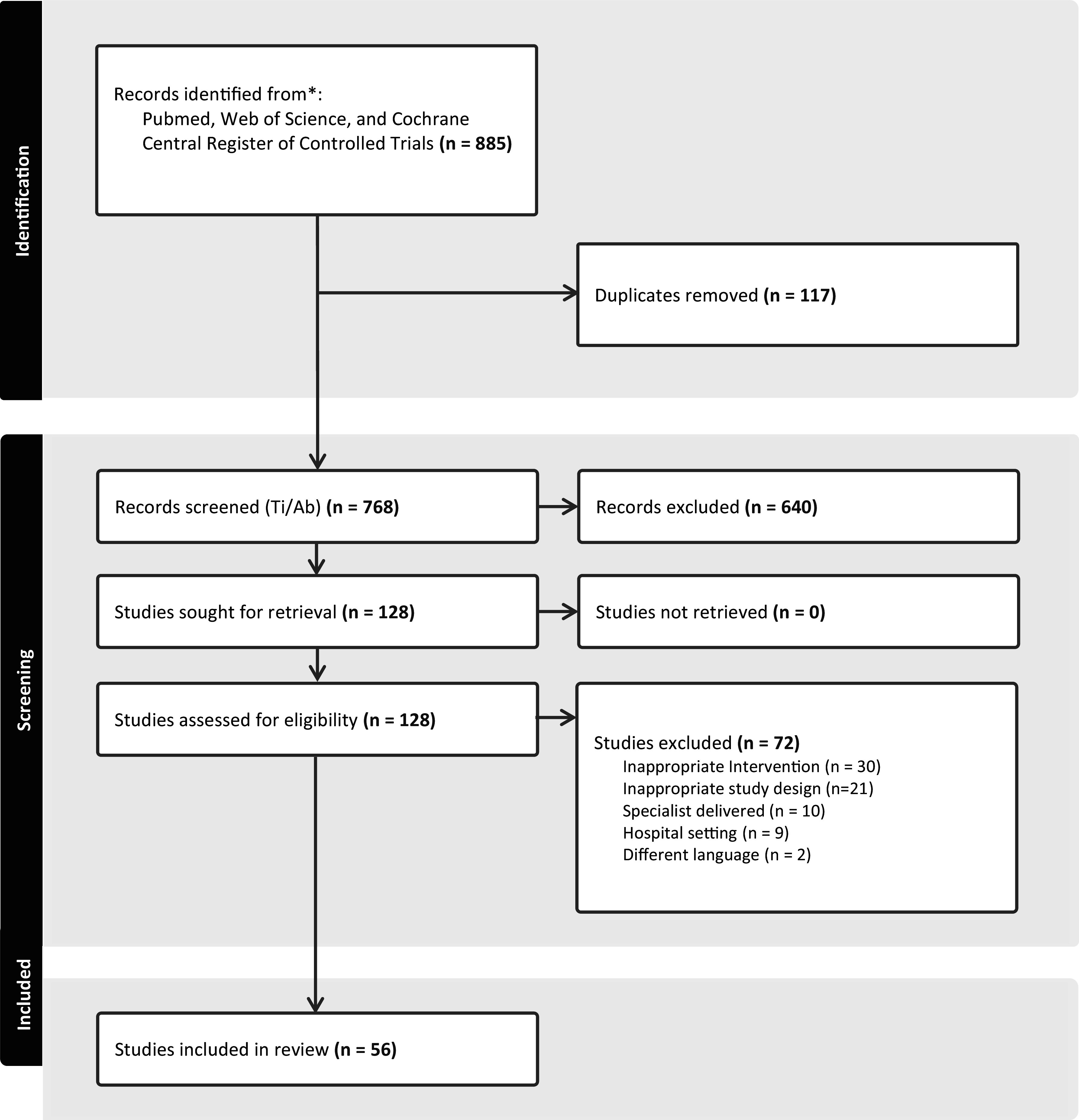
PRISMA flow diagram.

A total of 885 studies were retrieved, with 117 duplicates. After screening the titles/abstracts, 128 studies were reviewed in full and 56 met the inclusion criteria. Reasons for exclusion included inappropriate interventions, study design, specialist-delivered, hospital settings or language other than English (see Figure 1).

### Quality assessment

Altogether, 15 qualitative studies were assessed based on the CASP checklist. While most qualitative studies provided clear objectives, methodology and findings, there was inadequate reporting on the researcher’s positionality (60%), the value of the research (46.66%) and ethical issues (40%). A few studies focused on process documentation (Eappen et al., 2018; Yator et al., 2021), adaptation and development of interventions (Zayas et al., 2004); therefore, the study methods and data analysis were not applicable.

Overall, studies employing quantitative and mixed methods (n = 41) had adequately described their purpose (n = 39), interventions (n = 38) and study methods (n = 34). Implementation outcomes as per Proctor’s framework were reported partially by 36 studies with most examining feasibility, training and supervision outcomes. The included trials and pilot studies poorly reported on study team (n = 11), transparency of data analysis (n = 9) and protocol registration (n = 13) (see Supplementary File 2).

### Description of studies

Twenty-four studies were published in HICs, followed by LMICs (n = 23) and upper middle-income countries (n = 9). Most studies were quantitative (n = 30), followed by qualitative (n = 15) and mixed methods (n = 10). Studies ranged from intervention development to implementation and effectiveness testing. One study reported it as a prevention intervention (Zayas et al., 2004), but the reference article (Miranda and Muñoz, 1994) clarified that it targeted mild depression, justifying its inclusion. Details are given in Table 2.

**Table 2.** Study description and key characteristics of intervention

Author year	Methods			Intervention details						Delivery agents
	Country	Study design	Aims	Theoretical underpinning	Intervention target	Mode of delivery	Type	Number of sessions	Duration	Type of NSHW
VanLieshout 2020	Canada	Quantitative	To test feasibility and acceptability of training the nurses to deliver intervention	CBT	Postnatal depression	In-person	Group	Nine	120 min	Nurses
Posmontier 2016	USA	Quantitative	To test feasibility and acceptability of the intervention	IPT	Postnatal depression	Telephone	Individual	Eight	50 min	Nurses
Leocata 2021	India	Qualitative	Ethnographic study to explore fidelity using manuals to guide sessions	CBT	Perinatal depression	In-person	Individual	15	N/A	Peers
Boisits 2021	South Africa	Mixed methods	To describe intervention development process	CBT and problem-solving	Perinatal depression and anxiety	In-person	Individual	Three	45 min	Community Health Worker
Fuhr 2019	India	Quantitative	To assess the effectiveness and cost-effectiveness of THP	CBT	Perinatal depression	In-person	Individual	6–14	30–45 min	Community Health Worker
Munodawafa 2017	South Africa	Qualitative	To explore barriers and facilitators of task-sharing intervention	CBT, IPT and problems solving	Antenatal depression	In-person	Individual	Six to eight	N/A	Community Health Worker
Glavin 2010b	Norway	Quantitative	To examine effectiveness of the intervention	Non-directive counseling	Postnatal depression	In-person	Individual	Individualized	30 min	Nurses
Singla 2020	India	Mixed methods	To explore barriers and facilitators of peer supervision and validate tool	CBT	Perinatal depression	In-person	Individual	14	45 min	Peers
Dennis 2013	Canada	Mixed methods	To explore service providers’ experience delivering the intervention	Emotional and peer support	Postnatal depression	Telephone	Individual	Minimum four	Individualized	Peers
Ransing 2021	India	Qualitative	To describe intervention development process	Psychoeducation, relaxation, health promotion	Perinatal depression	In-person	Individual	Three	15–20 min	Community Health Worker
Craig 2005	Australia	Quantitative	To examine effectiveness of the intervention	CBT	Postnatal depression	In-person	Group	Nine	120 min	Community Health Worker
Rahman 2007	Pakistan	Qualitative	To explore barriers and facilitators developing and delivering intervention	CBT	Perinatal depression	In-person	Individual	16	45 min	Community Health Worker
Morrell 2009	England	Quantitative	To examine effectiveness of the intervention	CBT or non-directive counseling	Postnatal depression	In-person	Individual	Eight	60 min	Midwives/nurse
Eappen 2018	Peru	Qualitative	To describe preparatory steps before implementation	CBT	Perinatal depression	In-person	Individual	N/A	N/A	Community health worker
Dennis 2014	Canada	Mixed methods	To describe development and implementation process	Emotional and peer support	Postnatal depression	Telephone	Individual	Minimum four	Individualized	Peers
Husain 2021	Pakistan	Quantitative	To test efficacy of the intervention	Problem solving; psychoeducation and parenting	Maternal depression and parenting	In-person	Group	10	60–90 min	Community health worker
Letourneau 2011	Canada	Quantitative	To examine effectiveness of the intervention	Peer support	Postnatal depression	In person or through telephone	Individual	12	20 min	Peers
Özkan 2020	Turkey	Quantitative	To examine effectiveness of the intervention	Exercise	Postnatal depression	In-person	Mixed	Regularly for 4 weeks	N/A	Midwives/nurses
Slade 2010	England	Qualitative	To explore experience of postnatal women engaging in intervention	CBT or person- centered intervention	Postnatal depression	In-person	Individual	Up to eight	60 min	Midwives/nurse
Singla 2021	India and Pakistan	Quantitative	To explore mediators influencing effectiveness of intervention	CBT	Perinatal depression	In-person	Individual (India); Group (Pakistan)	14	Up to 45 min	Peers
Nyatsanza 2016	South Africa	Qualitative	To describe development process of manual	CBT, problem-solving	Perinatal depression	In-person	Individual	Six	N/A	Midwives
Nakku 2021	Uganda	Quantitative	To examine effectiveness of the intervention	Problem Solving	Perinatal depression	In-person	Group	Minimum four	90–120 min	Midwives
Roman 2009	USA	Quantitative	To examine effectiveness of the intervention	Problem-solving	Perinatal depression	In person or through telephone	Individual	Up to 18	N/A	Nurses
Dennis 2003	Canada	Quantitative	To explore feasibility and preliminary efficacy of intervention	Peer support	Postnatal depression	Telephone	Individual	N/A	N/A	Peers
Nisar 2020	China	Mixed methods	To adapt intervention and explore acceptability	CBT	Perinatal depression	N/A	N/A	Seven	N/A	Nurses
Russell 2020	USA	Qualitative	To explore users’ experience engaged in intervention	Motivational interviewing and mindful exercise	Antenatal depression	In-person	Group	12	75 min	Nurses
Zayas 2004	USA	Qualitative	To describe lessons learned during intervention implementation	CBT	Perinatal depression	In-person	Individual	Eight	N/A	Graduate students
Ross 2013	Thailand	Mixed methods	To examine effectiveness of the intervention	Emotional and informational support	Antenatal depression	Telephone	Individual	N/A	15–30 min	Nurses
Tezel 2006	Turkey	Quantitative	To examine the impact of the intervention	Problem solving	Postnatal depression	In-person	Individual	N/A	N/A	Nurses
Leocata 2021	India	Qualitative	To explore service providers’ experience delivering the intervention	CBT	Perinatal depression	In-person	Individual	N/A	N/A	Peers
Sawyer 2019	Australia	Quantitative	To examine effectiveness of the intervention	Maternal self-efficacy and social support mechanism	Postnatal depression and parenting problems	Virtual App based	Group	N/A	N/A	Nurses
Yator 2021	Kenya	Qualitative	To describe implementation process and acceptability of intervention	IPT	Postnatal depression	In-person	Group	8	N/A	Community health worker
Tryphonopoulos 2020	Canada	Quantitative	To test efficacy of the intervention	Barnard Model Maternal–infant interaction	Postnatal depression and maternal infant interaction	In-person	Mother–infant dyad	Three	60–90 min	Nurses
Chibanda 2014	Zimbabwe	Quantitative	To test efficacy of the intervention	Problem solving	Postnatal depression	In-person	Group	12	60 min	Peers
Nisar 2022	China	Quantitative	To examine effectiveness of electronic vs. face-to-face training	CBT	Perinatal depression	N/A	N/A	N/A	N/A	Nurses
Brock 2017	USA	Quantitative	To assess the sustainability of treatment effect	Problem solving	Antenatal depression	In-person	Individual	Six	30–50 min	Midwives/nurse
Notiar 2021	Kenya	Mixed methods	To test the acceptability and feasibility of the intervention	CBT	Maternal depression	In-person	Group	12	60–90 min	School teacher/priest
Layton 2020	Canada	Qualitative	To explore NSWHs’ experience delivering the intervention	CBT	Postnatal depression	In-person	Group	Nine	120 min	Nurses
Carter 2020	UK	Qualitative	To test feasibility and acceptability of the intervention	Peer support	Antenatal depression	In-person	Individual	Six	60 min	Peers
Sikander 2019	Pakistan	Quantitative	To examine effectiveness and cost-effectiveness of intervention	CBT	Perinatal depression	In-person	Mixed	14	N/A	Peers
Horowitz 2013	United States	Quantitative	To test efficacy of the intervention	CBT	Postnatal depression and mother and child bond	In-person	Individual	Two	30–40 min	Nurses
Appleby 2003	UK	Quantitative	To test acceptability of training the non-specialists	CBT	Postnatal depression	N/A	N/A	N/A	N/A	Midwives/nurse
Rahman 2008	Pakistan	Quantitative	To test effectiveness of the intervention	CBT	Perinatal depression and nutrition	In-person	Individual	16	N/A	Community health worker
Gureje 2019	Nigeria	Quantitative	To test effectiveness of the intervention	Problem solving	Perinatal depression	In-person	Individual	8–16+	N/A	Community health worker
Prendergast 2001	Australia	Quantitative	To test feasibility of training the non-specialists and its impact	CBT	Postnatal depression and anxiety	In-person	Individual	Six	60 min	Nurses
Dennis 2020	Canada	Quantitative	To test effectiveness of the intervention	IPT	Postnatal depression	Telephone	Individual	12	60 min	Nurses
Yator 2021	Kenya	Quantitative	To test feasibility and acceptability of the intervention	IPT	Postnatal depression	In-person	Group	N/A	N/A	Community health worker
Amani 2021	Canada	Quantitative	To test effectiveness of the intervention	CBT	Postnatal depression	In-person	Group	Nine	120 min	Peers
Rahman 2019	Pakistan	Quantitative	To examine effectiveness of electronic vs. face-to-face training	CBT	Perinatal depression	N/A	N/A	N/A	N/A	Community health worker
Atif 2019	Pakistan	Mixed methods	To describes the process of training and supervision of NSHW	CBT	Perinatal depression	N/A	N/A	N/A	N/A	Community health worker
Tomlinson 2020	Afghanistan	Mixed methods	To test feasibility of training NSHWs for intervention delivery	CBT	Postnatal depression	In-person	Individual	N/A	N/A	Midwives
Atif 2019	Pakistan	Mixed methods	To describe NSHWs experience after project completion	CBT	Maternal depression	In-person	Group	18+	N/A	Peers
Ng’oma 2019	Malawi	Qualitative	To describe intervention development process	N/A	Perinatal depression	N/A	N/A	N/A	N/A	N/A
Atif 2016	Pakistan	Qualitative	To explore NSHWs’ perceived feasibility and acceptability of intervention delivery prior to implementation	CBT	Perinatal depression	In-person	Group	N/A	N/A	Peers
Dennis 2010	Canada	Quantitative	To describe experiences of women engaged in intervention	Peer support	Postnatal depression	Telephone	Individual	Individualized	Individualized	Peers
VanLieshout 2022	Canada	Quantitative	To examine effectiveness of group intervention	CBT	Perinatal depression	In-person	Group	Nine	120 min	Nurses

Abbreviations: CBT, cognitive behavioral therapy; IPT, interpersonal therapy; N/A, not available.

### Implementation process

#### Intervention details

Most interventions targeted postnatal depression (n = 26), followed by perinatal (n = 22), antenatal (n = 5) and maternal depression (n = 3). Anxiety (Prendergast and Austin, 2001; Boisits et al., 2021), parenting (Sawyer et al., 2019; Husain et al., 2021), infant development (Zayas et al., 2004) and mother–infant relationship (Horowitz et al., 2013; Atif, Bibi, et al., 2019) were also addressed in some interventions. Cognitive behavioral therapy was the most widely used approach (n = 28), followed by problem-solving therapy (n = 5) and interpersonal therapy (IPT) (n = 4). Telephone-based interventions provided peer support (n = 4), psychoeducation (n = 1) or IPT sessions (n = 2). Most interventions (six studies missing information) were delivered in-person at home, health facilities or community centers (n = 40), followed by remote (n = 8) and hybrid sessions (n = 2). Session lasted between 15 min and 2 h, with individual sessions generally being shorter. Refer to Table 2 for further details.

#### Delivery agents and their characteristics

Nurses/midwives (n = 24) were the most common cadres, followed by peers (n = 15), community health workers (CHWs) (n = 14), school teachers/local priests (n = 1) (Notiar et al., 2021) and graduate students (n = 1) (Zayas et al., 2004). One study on intervention development did not mention the occupations of the NSHWs (Ng’oma et al., 2019). Nurses/midwives typically held diplomas or master’s degrees or had extensive nursing experience, but no mental health training. Peers in LMICs were local married women sharing similar culture and socioeconomic status (Atif, Bibi, et al., 2019; Atif, Nisar, et al., 2019; Fuhr et al., 2019; Rahman et al., 2021). Peers in HICs were matched by lived experience of perinatal depression (Dennis, 2003, 2010, 2013, 2014; Letourneau et al., 2011; Amani et al., 2021). In Zimbabwe, health facilities providing prevention services for mother-to-child HIV transmission trained and mobilized HIV-infected women as peer counselors (Chibanda et al., 2014). CHWs were often local females, with at least secondary education and 2.5 years of work experience in maternal and child health programs.

#### Training

Forty-three studies reported details on the training for the NSHWs, while information from two studies (Brock et al., 2017; Sawyer et al., 2019) were obtained from secondary publications (Segre et al., 2015). Training duration ranged from 4 h to 2weeks, some with follow-up sessions and refresher training. Lectures, audiovisuals and discussions were common methods used for theoretical content delivery, alongside role play, session observation (Dennis et al., 2020; Layton et al., 2020) or internships (Chibanda et al., 2014; Atif, Bibi, et al., 2019; Fuhr et al., 2019) to enhance skills. The use of technology such as telephones and tablets for training was also described in some studies (Dennis, 2003, 2010, 2013, 2014; Rahman et al., 2019; Nisar et al., 2020; Nisar et al., 2022). Training content focused on the assessment and treatment of mental health conditions based on a structured manual/protocol and was usually delivered by psychiatrists, psychologists or specialists.

#### Supervision

The majority of the studies (n = 35) reported on supervision, with details of two studies retrieved (Brock et al., 2017; Leocata, Kleinman, et al., 2021) from secondary publications (Singla et al., 2014; Segre et al., 2015; Atif et al., 2017). Supervision primarily occurred face-to-face in-group settings on a weekly (n = 10), fortnightly (n = 1) or monthly (n = 11) basis or by need (n = 3) (Craig et al., 2005; Van Lieshout et al., 2020; Ransing et al., 2021). Electronic mediums such as telephones (Morrell et al., 2009; Dennis, 2013, 2014; Posmontier et al., 2016; Dennis et al., 2020), emails (Dennis, 2014) and apps (Eappen et al., 2018; Atif, Nisar, et al., 2019; Rahman et al., 2019; Yator et al., 2021) were also utilized. Supervision details (duration, frequency or content) were missing in nine studies (Slade et al., 2010; Letourneau et al., 2011; Carter et al., 2020; Layton et al., 2020; Leocata, Kleinman, et al., 2021; Leocata, Kaiser, et al., 2021; Notiar et al., 2021; Singla, MacKinnon, et al., 2021; Van Lieshout et al., 2022). Supervisors were predominantly mental health professionals, although peer-led supervision was common in studies involving peers as NSHWs. Some studies (n = 6) adopted a cascade model, where experts supervised local trainers who then supervised implementers (Atif et al., 2016; Atif, Bibi, et al., 2019; Atif, Nisar, et al., 2019; Rahman et al., 2019; Sikander et al., 2019; Leocata, Kleinman, et al., 2021). Supervision sessions mainly focused on reviewing intervention content, followed by practice sessions through role play, discussion on challenges faced during service delivery and potential strategies to manage burnout.

### Implementation outcomes based on Proctor’s framework

An overview of the outcomes is provided in Table 3.

**Table 3.** Implementation outcomes as per the Proctor’s Framework

Study ID	1. Feasibility	2. Acceptability	3. Appropriateness	4. Adoption	5. Fidelity	6. Cost	7. Penetration	8. Sustainability
VanLieshout 2020	Screening tool- Edinburgh Postnatal Depression Scale (EPDS) 7/12 were enrolled; 71.43% treatment adherence 100% response rate Service providers training completion rate– 100%	Childcare facility facilitated to attend sessions	Group sessions helped in normalization and strengthened bonding	N/A	N/A	N/A	N/A	N/A
Posmontier 2016	Screening tool- Hamilton Rating Scale for Depression (HAM-D)	N/A	Phone based sessions reduced stigma, improved comfort and saved transportation hassle	N/A	Method: Audio recording Raters: IPT Supervisors, experts Result: Not mentioned (methods only)	N/A	N/A	N/A
Leocata 2021	N/A	N/A	N/A	N/A	Method: Therapy Quality Scale Raters: N/A Result: N/A	N/A	N/A	N/A
Boisits 2021	Screening tool- EPDS Recruitment– 19.4% Follow up rate at 3 months– 93.5% for intervention and 80.4% for control Intervention completion– 53.3%	Metaphors, brief sessions eased service delivery. **Negative experience-NSHWs Inconsideration, manual reading, giving high hopes Positive experience- Psychoeducation and normalization, space to share, connection with NSHW.**	Stepwise approach was helpful. Need identified for more time, training and supervision.	Brief intervention (3 sessions) was easy to adopt by NSHWs	N/A	N/A	N/A	N/A
Fuhr 2019	Screening tool- Patient Health Questionnaire (PHQ– 9) 84·1% consented to participate; Treatment completion rate– 71·7%. 11·5% were discontinued and referred to specialist at 6 months	N/A	**Family member engagement, and need to address incentives**	N/A	Method: Audio recording rated on Therapy Quality Scale Raters: N/A Result: N/A	Mean cost: USD 1.36 (12% attributed to incentives)	N/A	N/A
Munodawafa 2017	47.8% completed six sessions. 27.2% dropped out 26.7% women did not complete all 6 sessions.	**Secondary article- Lack of empathy, reading from the manual, giving high hopes were barriers while normalization, strong bond, hope for change, having a space to share problems were facilitators.**	N/A	Challenges negotiating the clinic environment. Not welcomed by the health facility staff stood as a barrier	Method: Audio recording Raters: Supervisor Result: 62.8% fidelity rating meaning moderate to good adherence to the manual	N/A	N/A	N/A
Glavin 2010b	Screening tool- EPDS **Response rates at t2 (3 months)– 85.5%, at t3 (6 months)– 66.3%, t4 (12 months postpartum)– 54.2%**	**Training elevated their confidence to support. Screening was easy and made it feasible to integrate in daily lives.**	N/A	N/A	N/A	N/A	N/A	N/A
Singla 2020	N/A	N/A	N/A	Lack of compensation; lack of time, other commitments impeded implementation	Method: Audio recording (Rating tool- Therapy Quality Scale) Raters: Peers Result: N/A	N/A	N/A	N/A
Dennis 2013	N/A	Motivators- personal growth, knowledge and social network expansion, altruism,	N/A	N/A	N/A	N/A	N/A	N/A
Ransing 2021	Nurses were able to screen without difficulty post-training. Nearly 50% suggested 2–3 sessions to reduce attrition	**Secondary article – 83% mothers found the intervention useful and that it contributed to their learning**	A quarter midwives expressed extra workload and suggested monetary incentivization for motivation	N/A	N/A	N/A	N/A	N/A
Craig 2005	Screening Tool- Hospital Anxiety and Depression Scale (HADS) Response rates– 14/16 completed pre-post data at 6 weeks follow up– 7 (50%); 6 months follow up – 7 (50%)	N/A	N/A	N/A	N/A	N/A	N/A	N/A
Rahman 2007	N/A	48% satisfied with the intervention	NSHWs found training relevant and easy to understand	N/A	N/A	N/A	N/A	N/A
Morrell 2009	Screening tool- EPDS 53% with a live baby consented to take part.	N/A	N/A	N/A	N/A	N/A	N/A	N/A
Eappen 2018	Screening tool- Self-Reporting Questionnaire (SRQ–18)	N/A	N/A	Longer intervention difficult to adopt; difficult geography hindered accessibility	N/A	N/A	N/A	N/A
Dennis 2014	Screening tool- EPDS Peer volunteers supported 2 mothers	Easy to maintain activity log. NSHWs felt supported and were willing to engage. Majority of mothers were satisfied, felt peer was helpful	N/A	N/A	Method: Intervention Protocol Raters: N/A Result: N/A	N/A	N/A	N/A
Husain 2021	Screening tool- PHQ–9 Response rates above 90% at 3 timepoints	N/A	N/A	N/A	Method: Observation Raters: Independent senior researchers Result: N/A	N/A	N/A	N/A
Letourneau 2011	Screening tool- EPDS Recruitment – Only 65 could be recruited Hospitalizations, relocation, lack of time and loss of contact led to incomplete data	N/A	N/A	N/A	N/A	N/A	N/A	N/A
Özkan 2020	Screening tool- EPDS	N/A	N/A	N/A	N/A	N/A	N/A	N/A
Slade 2010	Screening tool- EPDS Recruitment– 6/39 declined; 3 did not respond	Barriers: sessions as informal, intervention not tailored. Facilitators- space to discuss changes	N/A	N/A	Method: Audio recording Raters: Not mentioned Result: Good adherence	N/A	N/A	N/A
Singla 2021	Screening tool- PHQ–9	N/A	N/A	N/A	N/A	N/A	N/A	N/A
Nyatsanza 2016	Screening tool – EPDS	Perceived acceptability of counseling intervention by both service users and delivery agent but 33% were fearful to share problems.	Local female counselors, monthly individual sessions perceived appropriate. 50% reported work burden.	N/A	Method: Observation during supervision and feedback Raters: N/A Result: N/A	N/A	N/A	N/A
Nakku 2021	Screening tool- PHQ–9 20/30 midwives completed participation. Drop-out due to transfer and heavy workloads. 98.3% consent rate. 90.2% response rate	N/A	N/A	N/A	N/A	N/A	N/A	N/A
Roman 2009	Screening tool- Center for Epidemiological Studies Depression (CES-D) NSHWs able to engage high risk women. 82% participated in the intervention (18% miscarriage, moved out of the county or withdrew).	N/A	N/A	N/A	Method: Activity log (Clinical records) Raters: Senior team member (nurse) Result: N/A	N/A	N/A	N/A
Dennis 2003	Screening tool- EPDS 67% consented participation. 80% response rate Average login– 5 or more actual connections and 5 attempted connections for most mothers	NSHWs were satisfied with the helping experience Mothers were satisfied, felt that they received emotional, informational and appraisal support,	Perceived NSHW as trustworthy and were willing to engage in the future.	N/A	Method: Activity logs used Raters: N/A Result: N/A	N/A	N/A	N/A
Nisar 2020	Screening tool- PHQ–9 96 out of 100 students (96%) completed the assessment.	THP sessions helpful, acceptable, appropriate and meaningful Concepts clear and understandable and culturally relevant	Nurses identified as most appropriate cadre for intervention delivery	N/A	N/A	N/A	N/A	N/A
Russell 2020	Screening tool- PHQ–9 **82% enrollment rate 66% completed 12 week intervention; Drop out reasons: no longer pregnant, move out of area, side effects; 9 were lost to follow up**	Group intervention provided “safe space,” built “sisterhood bond,” “shared experiences with other participants,” helped in “normalization,” learned coping mechanisms.	N/A	N/A	N/A	N/A	N/A	N/A
Zayas 2004	Screening tool- Beck’s Depression Inventory (BDI) 38% attrition. Retention was a challenge in studies of urban, minority poor populations.	N/A	NSHWs preferred health facility over home visits for service delivery citing safety concerns.	Logistical challenges: frequent turnover of NSHWs were noted challenges	Method: N/A Raters: N/A Result: N/A Mention of challenges not assessing fidelity	N/A	N/A	N/A
Ross 2013	Screening tool- CES-D	NSHWs availability, quality of relationship, emotional support led to higher acceptability.	Telephone based support although helpful concerns of confidentiality	N/A	N/A	N/A	N/A	N/A
Tezel 2006	Two steps screening using EPDS and BDI **4/70 declined participation, 3 moved to other district, 1 denied participation in the study by her spouse.** **Recruitment rate– 88.57%**	N/A	N/A	N/A	N/A	N/A	N/A	N/A
Leocata 2021	**NSHWs retention– 77.27% Average session attendance– 7.7/10;** **India- total sessions 2–4 Barriers to recruitment- Refusal, migration** **Barriers to retention- uncontactable, withdrawal, drop out, visit to maternal home**	NSHW motivation- Personal development; Easy and helpful content; **adequate** t**raining and supervision,** elevated **social status, their chances of employment; improvements in women and their bonding with child**	**Matching characteristics facilitated discussion better. Volunteerism was acceptable in Pakistan was incentives expected in India.**	N/A	N/A	N/A	N/A	Concerns of NSHWs and service recipients on abrupt end of the program, which could not continue beyond the study’s timeframe.
Sawyer 2019	Screening tool- EPDS Uncontactable (mobile population) barrier to follow-up- App login rate– 50–60% Higher in week 1 and eventually lessened	Chatpage mostly used, moodtracker least used function. App perceived informative and resourceful,	N/A	N/A	N/A	N/A	N/A	N/A
Yator 2021	Screening tool- EPDS **25/32 consented to participate.** **Flexibility to mobilize NSHWs for follow up**	**Improved emotional and behavioral wellbeing, communication, empowerment, interpersonal skills, anger management, instilled hope.**	**NSHWs mediation role appreciated, Adequate training and supervision. NSHWs able to integrate in daily work. Ripple effect of intervention**	Lack of remuneration for NSHW affected the consistent delivery of regular sessions	Method: Audio recording/ Interpersonal inventory rating scale Raters: N/A Result: Younger NSHWs had a better understanding	N/A	N/A	N/A
Tryphonopoulos 2020	Screening tool- EPDS 63% able to recruit from potential participant referrals received from community recruitment partners	N/A	N/A	N/A	N/A	N/A	N/A	N/A
Chibanda 2014	Screening tool- EPDS 95% attendance rate; 85% treatment completion. 15% women were lost to follow-up with more from pharmacotherapy group.	N/A	N/A	N/A	N/A	N/A	N/A	N/A
Nisar 2022	96% post-training assessment completion rate.	E-training more acceptable, useful for learning, training	N/A	N/A	N/A	N/A	N/A	N/A
Brock 2017	Screening tool- Hamilton Rating Scale for Depression (HRSD) **52.8% excluded or denied services** **Reasons for exclusion- language barriers, not wanting to engage in the study** **6/66 did not complete assessment; Reasons- inability reach, no longer interested, referral, transferred out of program**	**Few wanted medication alongside intervention NSHW perceived as caring Intervention was “very helpful.” Impact- improved basic helping skills and problem-solving skills. 42% recommended more and/or longer sessions.**	**Participants said they would not change anything about where intervention took place or by whom it was delivered**	N/A	N/A	N/A	N/A	Sustainability of treatment effects especially in low-income women and mothers of young children.
Notiar 2021	Screening tool- PHQ–9 17/19 agreed to participate 59% completed 12 session treatment Reasons for nonadherence- full time jobs, large groups	Satisfaction with the support. It helped gain knowledge, wellbeing and coping. Willing to recommend the support	All families allowed them to attend the intervention, and most of them attended all sessions.	N/A	N/A	N/A	N/A	N/A
Layton 2020	All six NSHWs completed training and volunteered to participate in the study	Training was adequate, helpful and wholesome. Placements helped to practice skills. Supervision enhanced understanding Learning embedded in daily lives and led to personal development	N/A	N/A	N/A	N/A	N/A	Fear of intervention ending Feelings of concern and anxiety leading up to the completion of the support visits (n = 4)
Carter 2020	Screening tool- Whooley questionnaire	NSHWs were appreciated for nonjudgmental attitude, time and empathy. NSHWs gratified to deliver intervention but dissatisfied over brief intervention.	N/A	N/A	N/A	N/A	N/A	N/A
Sikander 2019	Screening tool- PHQ–9 78% completion rate Adherence- mean of 3·7/5 antenatal and a mean of 7·3/9 postnatal sessions completed 80% response rates	**Elevated social status as NSHWs**	**Family member engagement led to higher buy-in from family.** **Incentives motivated and sustained NSHWs**	N/A	Method: Observation Group classroom and field supervision Raters: Trainers Result: N/A	Mean cost: The THPP intervention cost US$133 per participant to deliver.	N/A	N/A
Horowitz 2013	Screening tool- EPDS	Intervention as a source of support; Video feedback made aware and reflect on actions.	N/A	N/A	N/A	N/A	N/A	N/A
Appleby 2003	N/A	Training was practical, useful and relevant leading to changes in knowledge, skills and competence post training	N/A	N/A	N/A	Pre training cost GBP 81, 116, 107 and post-training cost GBP 79, 108, 109 per patient, per depressed patient and per treated patient	N/A	N/A
Rahman 2008	Screening tool- HRSD Recruitment method- Official register Drop out conditions- abortion, still born, premature or congenitally disabled born, infant death, given up for abortion, seriously ill mothers, migration.	N/A	N/A	N/A	N/A	N/A	N/A	N/A
Gureje 2019	Screening tool- EPDS Lost to follow-up reasons- relocation or no contact 61% treatment completion rate	N/A	N/A	N/A	Method: Activity log (Clinical records) and direct observation Raters: Senior researchers Result: 58% were rated very good, 32% good and 10% poor	Mean cost: USD 46.85 per participant over 1 year in intervention arm vs. USD 24 in control	N/A	N/A
Prendergast 2001	Screening tool- EPDS All completed 6 sessions. Dropouts were higher in control group	70% of women in the intervention arm felt that the improvement was because of their NSHWs.	Health facility-based sessions (control arm) faced challenges relating to convenience, time arrangement and lack of concern on the mother.	N/A	Method: Audio recording Raters: Senior member (registrar) and psychiatrist Result: 70% of adhered to the protocol (problem-solving techniques and pleasant-event scheduling).	N/A	N/A	N/A
Dennis 2020	Two step screening process with EPDS followed by Structured Clinical Interview for DSM-IV (SCID-I) Recruitment rate– 93.8% Response rate– 81.7% Treatment adherence– 86.7% Reasons for nonadherence listed	Facilitators- Convenience, competent NSHWs, satisfaction with the sessions 58.2% wished for more sessions	N/A	N/A	Method: Checklist and activity logs; Audio recording Raters: IPT experts Result: High treatment fidelity (86%)	N/A	N/A	N/A
Yator 2021	Screening tool- EPDS	N/A	N/A	N/A	Method: Interpersonal inventory rating scale Raters: N/A Result: The mean score increased when IPT-G was offered to the waitlist control group.	N/A	N/A	N/A
Amani 2021	Screening tool- EPDS Above 50% response rates 84% intervention adherence rates	N/A	N/A	N/A	N/A	N/A	N/A	N/A
Rahman 2019	75% completed the assessment.	N/A	N/A	N/A	N/A	Conventional training cost- USD 170 per NSHW Technology assisted: USD 117 per NSHW	N/A	N/A
Atif 2019	75% NSHWs retained Dropout reasons- poor competency; migration; poor acceptance	Attendance in supervision sessions was above 85–100% throughout 3 years.	N/A	Linking to the health system led to greater buy-in from the community	N/A	N/A	N/A	N/A
Tomlinson 2020	Screening tool- PHQ–9 Only 55% participated in 65% adherence rate. Reasons for missing sessions- child illness, household commitments, prohibition by family, dissatisfaction	N/A	N/A	N/A	N/A	N/A	N/A	31/ 45 peer volunteers retained over 5 years.
Atif 2019	N/A	Motivators – altruism, social network expansion, personal growth gained respect and strong supervision	Supervision appropriate to prepare for helping role	Logistical and coordination challenge to conduct group session	N/A	N/A	N/A	N/A
Ng’oma 2019	Screening tool- EPDS	N/A	N/A	Barriers- busy and full clinics; no dedicated person, no guidelines.	N/A	N/A	N/A	N/A
Atif 2016	Screening tool- PHQ–9	Intervention was helpful which led to acceptability and motivation for NSHW.	N/A	N/A	N/A	N/A	N/A	N/A
Dennis 2010	Screening tool- EPDS 63.3% response completion	Intervention alleviated stress, promoted coping, social integration Satisfied with the support, Found convenient, accessible Strong positive bonding with the NSHWs.	N/A	N/A	N/A	N/A	N/A	N/A
VanLieshout 2022	Screening tool- EPDS Recruitment through social media, referral from the health facility. 33% lost to follow-up	N/A	N/A	N/A	N/A	N/A	N/A	N/A

Note: Texts written in **bold** were extracted from secondary article.

Abbreviations: BDI – Beck’s Depression Inventory; CES-D – Center for Epidemiologic Studies Depression; EPDS – Edinburgh Postnatal Depression Scale; IPT – Interpersonal Therapy; N/A – Not available; NSHW – Nonspecialist health worker; PHQ-9 – Patient Health Questionnaire-9; THP – Thinking Healthy Program.

#### Feasibility of interventions

Proctor’s framework defines feasibility in terms of recruitment, retention and adherence to treatment. Altogether, 32 studies reported feasibility outcomes. Additionally, seven secondary articles were reviewed to extract data on feasibility.

##### Recruitment and retention of service users

Recruitment of perinatal women primarily occurred at the health facility, but social media and advertisements were also utilized. Out of 43 studies that reported screening tools, Edinburgh Postnatal Depression Scale (EPDS) was the most common (n = 24), followed by the Patient Health Questionnaire (PHQ-9) (n = 10), Center for Epidemiological Studies Depression (n = 2), Hamilton Rating Scale for Depression (n = 2), Beck’s Depression Inventory (n = 2), Hospital Anxiety and Depression Scale (n = 1), Self-Reporting Questionnaire (n = 1) and Whooley’s questionnaire (n = 1). Studies were able to recruit between 67 and 94% of the total eligible women. A secondary article reported the lowest recruitment rate of 19% and cited language barriers, presence of comorbid conditions and experience of pregnancy loss as reasons for poor recruitment (Lund et al., 2020). Strict inclusion criteria often made recruitment a challenging and slow process (Letourneau et al., 2011), which was further exacerbated by unprecedented events such as COVID-19 (Amani et al., 2021).

Studies collecting data at multiple time-points generally had a 15–38% dropout at end line, but in some cases, dropout was as high as 91% (Husain et al., 2021). Retention was especially poor in studies that extended over 6 months in duration and studies involving urban minority low-income population (Zayas et al., 2004; Sawyer et al., 2019). Common reasons for poor retention were contact loss, hospitalization, time/interest constraints and program discontinuation.

##### Recruitment and retention of service providers

Five included articles (Roman et al., 2009; Atif, Nisar, et al., 2019; Van Lieshout et al., 2020; Nakku et al., 2021; Ransing et al., 2021) and one secondary article (Atif et al., 2017) reported the feasibility of recruiting, training and retaining the NSHWs. The feasibility of training and retaining NSHWs ranged from 67% to 100% (Roman et al., 2009; Van Lieshout et al., 2020; Nakku et al., 2021; Nisar et al., 2022). Common challenges pertaining to the retention of NSHWs included workload, transfer to different health facility, poor competency, migration, personal circumstances and poor acceptance by service users.

##### Service users’ adherence to treatment

The treatment completion rate ranged from 31 to 100%. A study conducted in Afghanistan had the lowest treatment participation and retention, citing household commitments, refusal from family, dissatisfaction and unavailability of health staff (Tomlinson et al., 2020). An individual-focused intervention that had six sessions delivered at home had a treatment completion rate as high as 100% (Prendergast and Austin, 2001). Adherence was higher (95%) in a health facility-based intervention when embedded within regular postnatal visits (Chibanda et al., 2014). For a telephone-based intervention, the treatment completion rate was as high as 98% once the treatment was initiated (Dennis et al., 2020). This was the opposite for an app-based intervention where the user engagement reduced over time (from 64% to 14% over 16 weeks) (Sawyer et al., 2019).

Postnatal sessions were frequently missed, partly due to the tradition of mothers returning to their maternal home for postnatal recovery. As this often involved relocation, home-based sessions became logistically challenging (Leocata, Kleinman, et al., 2021). One secondary article found as low as 28% attendance in postnatal sessions (Lund et al., 2020). Sickness, experiencing loss, lack of time, stigma, fear of breaking confidentiality and dissatisfaction with the services or NSHWs were cited as reasons for not engaging in care.

#### Acceptability

Twenty-four reviewed studies and seven secondary studies reported on acceptability.

##### Service providers

Self-driven, empathic and competent NSHWs were identified as key drivers to the intervention’s success. NSHWs delivering interventions in person or electronically reported positive experiences, viewing the intervention delivery as an opportunity to serve others and expand their social network (Singla et al., 2020). They also perceived that the training and intervention delivery experience enhanced their knowledge, skills and confidence, contributing to personal development (Appleby et al., 2003; Dennis, 2013; Glavin et al., 2010b; Layton et al., 2020; Boisits et al., 2021; Kukreti et al., 2022). Group supervision and tailored feedback helped address challenges and build confidence. Peer supervision, although beneficial, was less effective than expert supervision (Singla et al., 2020). Overall, NSHWs expressed satisfaction and willingness to engage in the future. Lack of confidence (Munodawafa et al., 2017; Carter et al., 2020), emotional burden (Dennis, 2013; Munodawafa et al., 2017) and resistance from family (Atif et al., 2016) hindered implementation.

Culturally appropriate content, illustrations and scripted guides better enabled NSHWs to deliver sessions (Dennis, 2014; Boisits et al., 2021; Leocata, Kaiser, et al., 2021). However, one study found that the violence-focused content was only beneficial for a specific demographic, suggesting its potential unsuitability as a universal intervention component (Ransing et al., 2021).

##### Service users

Engaging in the intervention yielded both physical and emotional benefits in service users. They expressed satisfaction with the NSHWs assigned to them. Educated, middle-aged females sharing similar language and culture were mostly preferred as NSHWs (Zayas et al., 2004; Singla et al., 2014; Nyatsanza et al., 2016). A strong match with the NSHW led to higher receptivity, trust and a strong bond (Dennis, 2010; Carter et al., 2020). Nurses were perceived as competent by 99% of the service users in one study (Dennis et al., 2020). However, NSHWs who read from manuals instead of engaging, did not give time, made invalidating remarks or set unrealistic hopes were seen as unhelpful (Slade et al., 2010; Davies et al., 2022).

Community-based health facility interventions were acceptable, and the provision of childcare eased attendance (Van Lieshout et al., 2020). Challenges included ill-equipped facilities and long waiting hours (Nyatsanza et al., 2016). Telephone-based support was accessible and alleviated concerns about transportation, time and childcare (Ross et al., 2013; Posmontier et al., 2016). For a mobile app-based intervention, a chat page where participants could communicate with NSHWs was the most used feature compared to a mood tracker or video content (Sawyer et al., 2019).

#### Appropriateness

Small group training with a mix of classroom-based and practical sessions was perceived as most beneficial by the NSHWs (Layton et al., 2020). Both electronic-based and in-person training were deemed useful (Rahman et al., 2019; Nisar et al., 2022). NSHWs felt that these trainings enhanced their knowledge, confidence and readiness for their role (Dennis, 2014; Russell et al., 2020; Yator et al., 2021).

Interventions complementing the existing system and tailored to contextual issues were deemed more appropriate (Nyatsanza et al., 2016; Ransing et al., 2021). NSHWs reported difficulty with issues outside the intervention’s focus (Munodawafa et al., 2017; Leocata, Kaiser, et al., 2021). Scripts provided structure, but some NSHWs found them constraining, highlighting a need for flexibility. Individual sessions allowed for discussing personal concerns and receiving tailored support (Slade et al., 2010), while group sessions fostered connections and normalized problems (Rahman, 2007; Russell et al., 2020; Van Lieshout et al., 2020). Service users preferred small groups and hesitated to engage in larger groups (Notiar et al., 2021).

Due to safety concerns and family resistance, home visits were less preferred by NSHWs (Zayas et al., 2004; Nyatsanza et al., 2016; Munodawafa et al., 2017; Leocata, Kleinman, et al., 2021). Phone- and app-based interventions were considered useful, user-friendly and less stigmatizing, but women reported discomfort receiving calls in others’ presence and missing each other’s calls (Dennis, 2010; Ross et al., 2013). The chat function in apps was particularly useful for asking questions (Sawyer et al., 2019).

For peers, incentives in the forms of financial payments, transportation and communication compensation, gifts or household items were cited as one of the key motivators for engaging in service delivery (Atif, Bibi, et al., 2019; Ng’oma et al., 2019; Sikander et al., 2019; Leocata, Kleinman, et al., 2021).

#### Programmatic adoption

Only nine studies reported on programmatic adoption. Brief interventions were easier to integrate into routine service at the health facility (Eappen et al., 2018; Boisits et al., 2021). Intervention delivery was easier for NSHWs when they linked their affiliation with the health facility (Atif, Nisar, et al., 2019). Health facility-based interventions had smooth functioning only when the health workers were cooperative. However, this placed an additional burden on NSHWs, requiring them to manage logistical, administrative and coordination tasks alongside providing psychological support (Zayas et al., 2004; Munodawafa et al., 2017; Atif, Bibi, et al., 2019). Lack of support from health facility staff (Zayas et al., 2004; Munodawafa et al., 2017), unequipped and inaccessible health facilities (Zayas et al., 2004; Eappen et al., 2018; Atif, Nisar, et al., 2019; Yator et al., 2021), lack of compensation and work burden (Atif, Bibi, et al., 2019; Atif, Nisar, et al., 2019; Ng’oma et al., 2019; Singla et al., 2020; Yator et al., 2021) hindered the implementation and adoption of the intervention in routine care. On the other hand, developing a maternal mental health guideline and creating a dedicated position within the health system were identified as facilitators for the integration of maternal mental health intervention into the health system (Ng’oma et al., 2019).

#### Fidelity

A total of 18 studies reported on fidelity. Fidelity was assessed through the rating of session observations or audio recordings, or activity logs using guidelines, checklists or tools such as the Therapeutic Quality Scale (Fuhr et al., 2019; Sikander et al., 2019; Singla et al., 2020; Leocata, Kaiser, et al., 2021) and Interpersonal Inventory Rating Scale (Yator et al., 2021). Fidelity assessments were mainly done to ensure adherence to the study protocol (Fuhr et al., 2019), intervention content, use of clinical skills (Prendergast and Austin, 2001; Nyatsanza et al., 2016; Munodawafa et al., 2017; Fuhr et al., 2019; Gureje et al., 2019) and to identify challenges leading to targeted training/supervision (Rahman, 2007; Munodawafa et al., 2017). Higher scores in these assessments meant higher fidelity to the intervention, while lower scores generally indicated a lack of competency to provide care. While many studies reported that NSHWs had good adherence to the intervention (Prendergast and Austin, 2001; Slade et al., 2010; Munodawafa et al., 2017; Gureje et al., 2019; Dennis et al., 2020), four studies reported challenges such as NSHWs lacking effective communication skills and struggling to adequately explain the intervention component or follow the manual (Eappen et al., 2018; Layton et al., 2020; Boisits et al., 2021; Davies et al., 2022).

#### Implementation cost

Altogether, five studies in the review reported cost analyses, of which three focused on the cost-effectiveness of the psychological intervention, whereas the other two focused on the training of NSHWs. Two studies reporting on the cost-effectiveness of the THP intervention in Pakistan and India reported that the intervention was highly cost-effective, with an estimation of $1 per beneficiary (Fuhr et al., 2019) and each unit of improvement on the PHQ-9 score costing between $2 and 20 (Sikander et al., 2019). Another study in Nigeria comparing high-intensity over low-intensity treatment found no difference in terms of cost effectiveness (Gureje et al., 2019). A study in the United Kingdom found that training NSHWs improved their skills and led to positive changes in their clinical practices without increasing the overall cost of service delivery (Appleby et al., 2003). Another study comparing the cost of technology-assisted training against in-person training found technology-assisted training more cost-effective by 30% (Rahman et al., 2019).

#### Penetration

Proctor’s framework defines penetration as a level of institutionalization and maintenance of treatment at the systems level, usually occurring in the mid to late stages of implementation. This information was missing in the reviewed studies.

#### Sustainability

Sustainability as institutionalization of treatment was not reported in the reviewed studies; however, four studies briefly outlined sustainability concerns. For example, engaging in short-lived projects affected NSHWs’ motivation to engage fully (Atif, Bibi, et al., 2019). Service users and their families expressed similar worries (Ross et al., 2013; Atif et al., 2016; Nyatsanza et al., 2016). One study reported treatment effects after 8 weeks (Brock et al., 2017), while another reported retention of peer volunteers (68.88%) over 5 years, suggesting the sustainability of local NSHWs (Atif, Bibi, et al., 2019).

## Discussion

There is a growing need for more evidence in implementation science, which focuses on translating theories into practice, identifying facilitators and barriers and developing strategies to overcome challenges (Rapport et al., 2018; Bauer and Kirchner, 2020). Qualitative insights to document the implementation process are essential, as they can serve as a guideline to practitioners aiming to integrate perinatal mental health in their programs. We applied Proctor’s framework of implementation science, which outlines implementation constructs and analyzes outcomes in the early, mid and late stages (Proctor et al., 2011), to report our findings. Our review found that most studies reported feasibility, acceptability, appropriateness and fidelity outcomes; however, very few evaluated cost, sustainability, adoption and penetration.

Our review indicates that acceptance and adherence were higher for interventions delivered at home or integrated in routine care when the NSHWs had matching characteristics with the service users. A strong bond with NSHWs was crucial, and without it, led to dissatisfaction with the program (Slade et al., 2010). For NSHWs, receiving training and supervision was a capacity-building opportunity, which enhanced their knowledge, confidence and readiness for the helping role (Dennis, 2014; Russell et al., 2020). None of the NSHWs had prior experience in mental health, therefore indicating the need for intensive training and supervision to maintain competency, ensure treatment quality, maintain fidelity and address emotional burnout (Watts et al., 2021). Incompetency of service providers can cause unintended harm (Dennis, 2010); hence, some studies in our review assessed competency when recruiting the NSHWs (Letourneau et al., 2011; Dennis, 2013, 2014; Munodawafa et al., 2017; Fuhr et al., 2019; Dennis et al., 2020; Singla et al., 2020; Singla, MacKinnon, et al., 2021). Evidence also highlights the need for competency-based training in mental health to ensure quality and safety of the treatment (Kohrt et al., 2020). A cross-country study in LMICs on Ensuring Quality in Psychological Support (EQUIP), an online platform to assess competency, found that competency-based training was helpful in reducing harmful behaviors and improving helpful behaviors of the NSHWs (Pedersen et al., 2023). Breuer et al. (2018) found that regular supervision motivated the NSHWs to proactively screen and manage mental health problems. While supervision dosage can vary, quality supervision is arguably more important than the quantity of supervision (Kemp et al., 2019).

Even when interventions are feasible, acceptable and effective, their adoption in the health system cannot be guaranteed (Bauer and Kirchner, 2020). Very few studies in this review reported on systems-level implementation outcomes, such as adoption or sustainability, and none reported on penetration. NSHWs were often in a voluntary position and were trained to integrate psychosocial intervention into their regular work. While there was a good receptivity of the intervention by the NSHWs, they expressed being demotivated and overburdened without incentives. Further, the temporary nature of these interventions raised concerns about their sustainability, often affecting the motivation of both the service providers and service users to engage in the intervention (Ross et al., 2013; Atif et al., 2016; Nyatsanza et al., 2016; Atif, Bibi, et al., 2019).

Poor adoption and sustainability of evidence-based treatments pose significant challenges to address maternal mental health (Bauer and Kirchner, 2020). While Proctor’s framework situates sustainability in the later implementation stages, emerging discourse suggests it is a continuous process spanning pre-, during and post-implementation phases (Pluye et al., 2004; Bergmark et al., 2018; Shelton et al., 2018). Program designers should proactively incorporate sustainability elements from inception, potentially through continuous stakeholder engagement to foster buy-in and cultivate an environment conducive to implementation. Strategies outlined by Vax et al. (2021) offer valuable guidance for implementing interventions.

Scaccia et al. (2015) emphasize the importance of assessing and ensuring “organizational readiness,” defined as having the willingness and capacity to implement the innovation for adoption and sustainability. Innovation that fits well with the needs, culture, context and capacity of the organization is more likely to be adopted (Scaccia et al., 2015; Vax et al., 2021). However, the pervasive stigma associated with mental health poses a threat to adoption. Structural stigma, marked by inadequate policies, political will and investment, limits service availability, (Jenkins et al., 2013; Livingston, 2020) while community-level stigma delays help-seeking and reduces service utilization (Livingston, 2020). Addressing stigma therefore requires innovative strategies at multiple levels. At the systems level, careful planning, funding and evidence-based advocacy – supported by cost-effectiveness studies – are essential for political buy-in and institutionalization within the health system (Bergmark et al., 2018; Vax et al., 2021). Meanwhile, at the community level, sensitization and engagement activities can foster awareness and encourage service uptake (Ng’oma et al., 2019; Subba et al., 2024).

The WHO’s guide for integration of perinatal mental health in maternal and child health services provides practical guidance for planners and policymakers on “what” actions can be taken to embed these interventions into routine care (WHO, 2022). However, a deeper understanding of “how” to implement these interventions in real-world settings and “what works and what does not” (including facilitators and barriers) remains essential. Despite the growing prominence of implementation science, the paucity of studies reporting process evaluation and implementation outcomes for perinatal depression interventions hinders the identification, replication and synthesis of evidence. Future studies could address this gap by using frameworks such as the Standard for Reporting Implementation Studies to report their findings, making them more visible and accessible (Pinnock et al., 2017).

## Limitations

The limitations of this review include the exclusion of non-English publications, which might have resulted in the omission of relevant articles. Second, this study conducted a narrative synthesis of the implementation constructs. For implementation constructs such as feasibility and fidelity that predominantly use quantitative measures, future research could consider conducting statistical analyses. Third, this review only focused on treatment interventions delivered by the NSHWs to the adult population. Given the wide engagement of NSHWs in prevention and promotion interventions globally and the focus across all age groups, this review could have excluded some important studies involving perinatal adolescents and girls.

## Conclusion

This review synthesized evidence on implementation outcomes using Proctor’s framework to gain insights into the process, success and barriers of NSHW-delivered psychosocial interventions. Findings indicate that such interventions are well-accepted, and NSHWs can effectively deliver them when adequately trained, supervised and incentivized. However, there is a notable lack of studies exploring systemic factors influencing adoption, maintenance and sustainability. Further research is needed to elucidate the factors affecting the systems level integration of these interventions. Future implementers would benefit from employing implementation science frameworks to guide planning, execution and sustainability while considering various implementation factors across different stages.

## Supporting information

Subba et al. supplementary material Subba et al. supplementary material

## Data Availability

The authors confirm that the data supporting the findings of this study are available within the article, references and/or its supplementary files.

## Abbreviations

CBT: cognitive behavioral therapy
HIC: high-income countries
HIT: high-intensity treatment
IPT: interpersonal therapy
LIT: low-intensity treatment
LMIC: low- and middle-income countries
NSHW: nonspecialist health workers
PRISMA: preferred reporting items for systematic review
WHO: World Health Organization

## Supplementary material

The supplementary material for this article can be found at http://doi.org/10.1017/gmh.2025.10010.
