# Supplementary material for: Feasibility and acceptability of community-based psychosocial interventions delivered by nonspecialists for perinatal common mental disorders: A systematic review using an implementation science framework
Source: Glob Ment Health (Camb). 2025 May 26;12:e54. doi: 10.1017/gmh.2025.10010 (PMC12186571; doi:10.1017/gmh.2025.10010)
Supplement: Subba et al. supplementary material [file S2054425125100101sup001.zip › Table S1 Search Strategy used in Cochrane Center Register of Controlled Trials.docx]

**Table S1: Search Strategy used in Cochrane Center Register of Controlled Trials**

| **Concepts** | **Key words/Query** |
| --- | --- |
| **Condition** | (antenatal OR antepartum OR pregnan* OR postnatal OR postpartum OR perinatal OR peripartum OR maternal):ti,ab,kw |
|  | (common NEXT (mental disorder* OR mental health problem*)):ti,ab,kw OR (depression):ti,ab,kw OR (depressive NEXT (disorder)):ti,ab,kw OR (depress*):ti,ab,kw OR (anxiety):ti,ab,kw OR (anxiety NEXT (disorder)):ti,ab,kw OR (anxi*):ti,ab,kw |
|  | MeSH descriptor: [Depression, Postpartum] explode all trees |
|  | MeSH descriptor: [Depression] explode all trees |
|  | MeSH descriptor: [Anxiety] explode all trees |
| **Psychological Interventions** | MeSH descriptor: [Psychotherapy] explode all trees |
|  | ((psychosocial NEXT (intervention* or counseling or counselling)):ti,ab,kw OR (psychoeducat*):ti,ab,kw OR (non NEXT (pharmacological or pharmaceutical)):ti,ab,kw OR (psychotherapy):ti,ab,kw OR (psychological or group NEXT (therapy)):ti,ab,kw OR (individual or group NEXT (counseling or counselling)):ti,ab,kw OR (psychoeducat*):ti,ab,kw OR (non NEXT (pharmacological or pharmaceutical)):ti,ab,kw OR (psychotherapy):ti,ab,kw OR (psychological or psychosocial NEXT (therapy or intervention)):ti,ab,kw OR (non directive NEXT (counseling or counselling)):ti,ab,kw OR (comprehensive or multifaceted or holistic or integrated NEXT (intervention)):ti,ab,kw OR (talk or relational or interpersonal NEXT (therapy)):ti,ab,kw OR (cognitive behavio* therapy):ti,ab,kw OR (dialectical behavio* therapy):ti,ab,kw |
| **Delivery Agents** | MeSH descriptor: [Community Health Workers] explode all trees |
|  | (psychosocial or "community health" or "lay health" or peer or health NEXT (worker or volunteer)):ti,ab,kw OR task NEXT (shar* or shift*):ti,ab,kw OR (nonspecialist):ti,ab,kw OR ("community health aide"):ti,ab,kw OR ("barefoot doctor"):ti,ab,kw OR (psychosocial or "community health" NEXT (counselor)):ti,ab,kw OR ("village health worker"):ti,ab,kw OR ("specially trained"):ti,ab,kw OR ("nurse"):ti,ab,kw |
|  | (implement):ti,ab,kw OR (practice):ti,ab,kw OR (reach*):ti,ab,kw OR (penetration):ti,ab,kw OR (train*):ti,ab,kw OR (sustainab*):ti,ab,kw OR (attitude*):ti,ab,kw OR (perception*):ti,ab,kw OR (behavior*):ti,ab,kw OR (facilitator*):ti,ab,kw OR (clinical NEXT (mentor* or supervis* or competen*)):ti,ab,kw OR (competen*):ti,ab,kw OR (barrier*):ti,ab,kw OR (acceptab*):ti,ab,kw OR (fidelity):ti,ab,kw OR (qualitative*):ti,ab,kw OR ("thematic analysis"):ti,ab,kw OR (feasibil*):ti,ab,kw OR (evaluation NEXT (stud* OR research*)):ti,ab,kw OR ("program* evaluation"):ti,ab,kw OR (ethnograph*):ti,ab,kw OR (phenomenology):ti,ab,kw OR ("content analysis"):ti,ab,kw OR (discourse*):ti,ab,kw OR ("grounded theory"):ti,ab,kw |
|  | MeSH descriptor: [Program Evaluation] explode all trees |
| **Limiter applied** | Jan 2000- Jan 2022 |
| **Results** | 734 [Search Date- 2 June 2022 (3:00 pm NPT)] |

**Table S2: Search strategy used in Web of Science Database**

| **Concepts** | **Key words Query (Search in AB and TI separately)** |
| --- | --- |
| Perinatal women | **(TI=((antenatal OR antepartum OR pregnan* OR postnatal OR postpartum OR "postpartum period" OR maternal OR perinatal OR peripartum) )) OR AB=((antenatal OR antepartum OR pregnan* OR postnatal OR postpartum OR "postpartum period" OR maternal OR perinatal OR peripartum) )** |
| Depression | **(TI=((depress* OR "depressive disorder" OR depression OR anxiety OR "anxiety disorder" OR anxi* OR "common mental disorder" OR "common mental health problem") )) OR AB=((depress* OR "depressive disorder" OR depression OR anxiety OR "anxiety disorder" OR anxi* OR "common mental disorder" OR "common mental health problem") )** |
| Psychological  Interventions | **(TI=(("psychosocial intervention" OR "psychosocial counseling" OR "psychological intervention" OR psychoeducat* OR "non-pharmacological" OR psychotherapy OR "psychological therapy" OR "group therapy" OR "group counseling" OR "individual counseling" OR "group session*" OR "nondirective counseling" OR comprehensive OR multifaceted OR integrated OR multicomponent OR multidimension* OR holistic OR "community based" OR "cognitive behavioral therapy" OR "Dialectical Behavior Therapy" OR "interpersonal therapy" OR "Interpersonal Psychotherapy"))) OR AB=(("psychosocial intervention" OR "psychosocial counseling" OR "psychological intervention" OR psychoeducat* OR "non-pharmacological" OR psychotherapy OR "psychological therapy" OR "group therapy" OR "group counseling" OR "individual counseling" OR "group session*" OR "nondirective counseling" OR comprehensive OR multifaceted OR integrated OR multicomponent OR multidimension* OR holistic OR "community based" OR "cognitive behavioral therapy" OR "Dialectical Behavior Therapy" OR "interpersonal therapy" OR "Interpersonal Psychotherapy"))** |
| Non-specialist | **(TI=((nonspecialist* OR task shar* OR task shift* OR "community health worker*" OR "lay health worker*" OR "peer volunteer*" OR "community volunteer*" OR "health worker*" OR volunteer* OR "barefoot doctor" OR "psychosocial worker*" OR "psychosocial counselor*" OR "specially trained" OR nurse* OR "village health worker*"))) OR AB=((nonspecialist* OR task shar* OR task shift* OR "community health worker*" OR "lay health worker*" OR "peer volunteer*" OR "community volunteer*" OR "health worker*" OR volunteer* OR "barefoot doctor" OR "psychosocial worker*" OR "psychosocial counselor*" OR "specially trained" OR nurse* OR "village health worker*"))** |
| Implementation  Outcomes | **(TI=((implement* OR practice OR reach OR penetration OR train* OR "Clinical Mentor*" OR "Clinical Supervision" Or "clinical competence" OR competen* OR Sustainab* OR Attitude* OR perception* OR view* OR behavior* OR facilitator* OR barrier* OR Qualitative* OR discuss* OR focus* group discussion OR interview* OR "thematic analysis" OR feasibil* OR acceptab* OR evaluat* OR "program* evaluation" OR ethnograph* OR phenomenology* OR "content analysis" OR discourse OR "grounded theory" OR fidelity))) OR AB=((implement* OR practice OR reach OR penetration OR train* OR "Clinical Mentor*" OR "Clinical Supervision" Or "clinical competence" OR competen* OR Sustainab* OR Attitude* OR perception* OR view* OR behavior* OR facilitator* OR barrier* OR Qualitative* OR discuss* OR focus* group discussion OR interview* OR "thematic analysis" OR feasibil* OR acceptab* OR evaluat* OR "program* evaluation" OR ethnograph* OR phenomenology* OR "content analysis" OR discourse OR "grounded theory" OR fidelity))** |
| Limiters | 2000.01.01-2022.01.31 |
| Results | **203** [6 June 2022; Time- 12:00 PM NPT] |
