# Supplementary material for: Feasibility and acceptability of community-based psychosocial interventions delivered by nonspecialists for perinatal common mental disorders: A systematic review using an implementation science framework
Source: Glob Ment Health (Camb). 2025 May 26;12:e54. doi: 10.1017/gmh.2025.10010 (PMC12186571; doi:10.1017/gmh.2025.10010)
Supplement: Subba et al. supplementary material [file S2054425125100101sup001.zip › Table S2 Search strategy used in Web of Science Database.docx]

**Table S2: Search strategy used in Web of Science Database**

| **Concepts** | **Key words Query (Search in AB and TI separately)** |
| --- | --- |
| **Perinatal women** | **(TI=((antenatal OR antepartum OR pregnan* OR postnatal OR postpartum OR "postpartum period" OR maternal OR perinatal OR peripartum) )) OR AB=((antenatal OR antepartum OR pregnan* OR postnatal OR postpartum OR "postpartum period" OR maternal OR perinatal OR peripartum) )** |
| **Depression** | **(TI=((depress* OR "depressive disorder" OR depression OR anxiety OR "anxiety disorder" OR anxi* OR "common mental disorder" OR "common mental health problem") )) OR AB=((depress* OR "depressive disorder" OR depression OR anxiety OR "anxiety disorder" OR anxi* OR "common mental disorder" OR "common mental health problem") )** |
| **Psychological**  **Interventions** | **(TI=(("psychosocial intervention" OR "psychosocial counseling" OR "psychological intervention" OR psychoeducat* OR "non-pharmacological" OR psychotherapy OR "psychological therapy" OR "group therapy" OR "group counseling" OR "individual counseling" OR "group session*" OR "nondirective counseling" OR comprehensive OR multifaceted OR integrated OR multicomponent OR multidimension* OR holistic OR "community based" OR "cognitive behavioral therapy" OR "Dialectical Behavior Therapy" OR "interpersonal therapy" OR "Interpersonal Psychotherapy"))) OR AB=(("psychosocial intervention" OR "psychosocial counseling" OR "psychological intervention" OR psychoeducat* OR "non-pharmacological" OR psychotherapy OR "psychological therapy" OR "group therapy" OR "group counseling" OR "individual counseling" OR "group session*" OR "nondirective counseling" OR comprehensive OR multifaceted OR integrated OR multicomponent OR multidimension* OR holistic OR "community based" OR "cognitive behavioral therapy" OR "Dialectical Behavior Therapy" OR "interpersonal therapy" OR "Interpersonal Psychotherapy"))** |
| **Non-specialist** | **(TI=((nonspecialist* OR task shar* OR task shift* OR "community health worker*" OR "lay health worker*" OR "peer volunteer*" OR "community volunteer*" OR "health worker*" OR volunteer* OR "barefoot doctor" OR "psychosocial worker*" OR "psychosocial counselor*" OR "specially trained" OR nurse* OR "village health worker*"))) OR AB=((nonspecialist* OR task shar* OR task shift* OR "community health worker*" OR "lay health worker*" OR "peer volunteer*" OR "community volunteer*" OR "health worker*" OR volunteer* OR "barefoot doctor" OR "psychosocial worker*" OR "psychosocial counselor*" OR "specially trained" OR nurse* OR "village health worker*"))** |
| **Implementation**  **Outcomes** | **(TI=((implement* OR practice OR reach OR penetration OR train* OR "Clinical Mentor*" OR "Clinical Supervision" Or "clinical competence" OR competen* OR Sustainab* OR Attitude* OR perception* OR view* OR behavior* OR facilitator* OR barrier* OR Qualitative* OR discuss* OR focus* group discussion OR interview* OR "thematic analysis" OR feasibil* OR acceptab* OR evaluat* OR "program* evaluation" OR ethnograph* OR phenomenology* OR "content analysis" OR discourse OR "grounded theory" OR fidelity))) OR AB=((implement* OR practice OR reach OR penetration OR train* OR "Clinical Mentor*" OR "Clinical Supervision" Or "clinical competence" OR competen* OR Sustainab* OR Attitude* OR perception* OR view* OR behavior* OR facilitator* OR barrier* OR Qualitative* OR discuss* OR focus* group discussion OR interview* OR "thematic analysis" OR feasibil* OR acceptab* OR evaluat* OR "program* evaluation" OR ethnograph* OR phenomenology* OR "content analysis" OR discourse OR "grounded theory" OR fidelity))** |
| **Limiters** | 2000.01.01-2022.01.31 |
| **Results** | **203** [6 June 2022; Time- 12:00 PM NPT] |
