# Supplementary material for: Feasibility and acceptability of community-based psychosocial interventions delivered by nonspecialists for perinatal common mental disorders: A systematic review using an implementation science framework
Source: Glob Ment Health (Camb). 2025 May 26;12:e54. doi: 10.1017/gmh.2025.10010 (PMC12186571; doi:10.1017/gmh.2025.10010)
Supplement: Subba et al. supplementary material [file S2054425125100101sup001.zip › Table S3 Quality Appraisal of Mixed Methods and Quantitative studies.docx]

**Table S3: Quality Appraisal of Mixed Methods and Quantitative studies**

|  |  | **Planning** | **Design and Conduct** | | | | **Reporting** | |
| --- | --- | --- | --- | --- | --- | --- | --- | --- |
| **Author Year** | **Methods** | **Degree of separation between outcome and process evaluation teams described** | **Clearly state their purpose** | **The intervention should be clearly described and causal assumptions clarified.** | **Justify choice of timing and methods** | **If applicable- transparently reports the process of data analysis blind to trial outcomes/ or post hoc** | **Clearly labelled implementation outcomes** | **Published a full report of evaluation components or a protocol paper** |
| VanLieshout 2020 | Pilot study | n/a | yes | yes | no | n/a | partially | no |
| Posmontier 2016 | Quantitative | yes | yes | yes | yes | n/a | partially | yes |
| Boisits 2021 | Mixed methods | no | yes | yes | yes | n/a | partially | n/a |
| Fuhr 2019 | RCT | n/a | yes | yes | yes | yes | partially | yes |
| Glavin 2010 | RCT | n/a | yes | yes | no | no | partially | no |
| Singla 2020 | Mixed Methods | n/a | yes | yes | yes | n/a | partially | n/a |
| Dennis 2013 | Mixed Methods | n/a | yes | yes | yes | n/a | partially | n/a |
| Craig 2005 | Pilot study | no | yes | yes | yes | no | partially | no |
| Morrell 2009 | RCT | no | yes | yes | yes | unclear | partially | no |
| Dennis 2012 | Mixed methods | n/a | n/a | n/a | n/a | n/a | partially | yes |
| Husain 2021 | RCT | n/a | yes | yes | yes | yes | partially | no |
| Letourneau 2011 | RCT | n/a | yes | yes | yes | yes | partially | no |
| Özkan 2020 | RCT | n/a | yes | yes | yes | no | no | yes |
| Singla 2021 | RCT | n/a | yes | yes | yes | yes | no | no |
| Nakku 2021 | Quantitative | n/a | yes | yes | yes | yes | partially | n/a |
| Roman 2009 | RCT | yes | yes | yes | yes | no | partially | no |
| Dennis 2003 | Pilot study | unclear | yes | yes | yes | no | partially | no |
| Nisar 2020 | Mixed methods | yes | yes | yes | yes | n/a | partially | n/a |
| Ross 2013 | Mixed methods | unclear | yes | yes | yes | n/a | partially | no |
| Tezel 2006 | Quantitative | unclear | yes | yes | yes | no | no | no |
| Sawyer 2019 | RCT | yes | yes | yes | yes | no | partially | yes |
| Tryphonopoulos 2020 | Pilot study | unclear | yes | yes | no | no | partially | no |
| Chibanda 2014 | RCT | n/a | yes | yes | no | no | partially | yes |
| Nisar 2022 | RCT | yes | yes | yes | yes | yes | partially | yes |
| Brock 2017 | Quantitative | yes | yes | yes | yes | no | no | no |
| Notiar 2021 | Mixed methods | yes | yes | yes | yes | n/a | partially | no |
| Sikander 2019 | RCT | yes | yes | yes | yes | yes | partially | yes |
| Horowitz 2013 | RCT | unclear | yes | yes | yes | no | partially | yes |
| Appleby 2003 | Quantitative | n/a | yes | yes | yes | n/a | partially | n/a |
| Rahman 2008 | RCT | yes | yes | yes | yes | no | no | yes |
| Gureje 2019 | RCT | unclear | yes | yes | yes | no | partially | yes |
| Prendergast 2016 | RCT | n/a | yes | no | no | no | partially | no |
| Dennis 2020 | RCT | yes | yes | yes | yes | no | partially | no |
| Yator 2021 | Pilot study | yes | yes | yes | yes | yes | partially | no |
| Amani 2022 | RCT | no | yes | yes | yes | no | partially | yes |
| Rahman 2019 | RCT | no | no | yes | yes | yes | partially | no |
| Atif 2019 | Mixed methods | n/a | yes | unclear | yes | n/a | partially | n/a |
| Tomlinson 2020 | Feasibility study | no | yes | yes | no | n/a | partially | no |
| Atif 2019 | Mixed methods | no | yes | yes | yes | n/a | partially | n/a |
| Dennis 2010 | Quantitative | no | yes | yes | yes | n/a | partially | n/a |
| VanLieshout 2022 | RCT | n/a | yes | yes | yes | no | partially | yes |
