# Supplementary material for: Feasibility and acceptability of community-based psychosocial interventions delivered by nonspecialists for perinatal common mental disorders: A systematic review using an implementation science framework
Source: Glob Ment Health (Camb). 2025 May 26;12:e54. doi: 10.1017/gmh.2025.10010 (PMC12186571; doi:10.1017/gmh.2025.10010)
Supplement: Subba et al. supplementary material [file S2054425125100101sup001.zip › Table S4 Quality Appraisal of Qualitative studies.docx]

**Table S4: Quality Appraisal of Qualitative studies**

| **Author, Year** | **Clear statement of aims** | **Appropriate methodology** | **Appropriate research design** | **Detailed, justified, recruitment strategy** | **Appropriate data collection methods** | **Researcher-participant relationship considered** | **Ethical issues considered** | **Rigorous data analysis** | **Clear findings** | **Value of research discussed** |
| --- | --- | --- | --- | --- | --- | --- | --- | --- | --- | --- |
| Leocata, Kaiser 2021 | yes | yes | yes | insufficient | insufficient | insufficient | insufficient | yes | yes | yes |
| Munodawafa 2017 | yes | yes | yes | yes | yes | yes | yes | yes | yes | yes |
| Ransing 2021 | yes | yes | yes | insufficient | yes | n/a | n/a | insufficient | insufficient | yes |
| Rahman 2007 | yes | yes | yes | insufficient | insufficient | yes | yes | yes | yes | yes |
| Eappen 2018 | yes | yes | n/a | n/a | n/a | n/a | n/a | n/a | n/a | n/a |
| Slade 2010 | yes | yes | yes | yes | yes | no | yes | yes | yes | insufficient |
| Nyatsanza 2016 | yes | yes | yes | yes | yes | no | yes | yes | yes | insufficient |
| Russell 2020 | insufficient | yes | yes | yes | yes | yes | yes | yes | yes | yes |
| Zayas 2004 | yes | n/a | n/a | yes | no | n/a | n/a | n/a | yes | yes |
| Leocata, Kleinman 2021 | yes | yes | yes | yes | yes | yes | insufficient | yes | yes | insufficient |
| Yator 2021 | yes | yes | n/a | n/a | n/a | n/a | n/a | insufficient | yes | insufficient |
| Layton 2020 | yes | yes | yes | yes | yes | yes | yes | yes | yes | insufficient |
| Carter 2020 | yes | yes | yes | yes | yes | yes | yes | yes | yes | yes |
| Ng'oma 2019 | yes | yes | yes | yes | yes | no | yes | yes | yes | yes |
| Atif 2016 | yes | yes | yes | yes | yes | no | yes | yes | yes | insufficient |
